# Safety and cost of selective histopathological analysis for detecting cancer in surgical specimens: a systematic review

**DOI:** 10.1111/ans.19380

**Published:** 2025-01-06

**Authors:** Angelique N. Camilos, Leo C. Bowley Schubert, Marcela P. Castro, Silas D. Nann, Suzanne Edwards, Brandon Stretton, Aashray K. Gupta, Joshua G. Kovoor, Matthew Marshall‐Webb, Guy J. Maddern

**Affiliations:** ^1^ Faculty of Health and Medical Sciences The University of Adelaide Adelaide South Australia Australia; ^2^ Department of Surgery, The Univeristy of Adelaide The Queen Elizabeth Hospital Adelaide South Australia Australia; ^3^ Department of Surgery Royal Adelaide Hospital Adelaide South Australia Australia; ^4^ Department of Cardiothoracic Surgery Gold Coast University Hospital Gold Coast Queensland Australia; ^5^ Adelaide Health Technology Assessment, School of Public Health The University of Adelaide Adelaide South Australia Australia

**Keywords:** diagnostic tests, routine, neoplasms, pathology, surgical, surgical procedures, operative, treatment outcome

## Abstract

**Background:**

Due to limited healthcare resources, there is global incentive to maximize efficacy while minimizing patient harm. Given the low rate of cancer diagnoses made via routine histopathological analysis of surgical specimens, a selective approach has been proposed as a viable alternative. This systematic review aimed to evaluate effectiveness of cancer detection and costs with a selective approach.

**Methodology:**

This study was registered with PROSPERO (CRD42022346535) and conducted according to PRISMA 2020 and MOOSE guidelines. Ovid Embase, Ovid MEDLINE and PubMed were searched from earliest result (1973) to 30 July 2022 for studies evaluating selective histopathology for surgical specimens. Screening, risk of bias assessment and data extraction were completed in duplicate. Statistical analysis used a random effects model.

**Results:**

Searches identified 4194 records, with 11 studies included consisting of 26 126 patients. Eight studies analysed patients who underwent cholecystectomy while three analysed patients who underwent appendectomy, vertical laparoscopic sleeve gastrectomy and neurectomy. In total, 295 neoplasms were detected: 196 malignant, 99 benign. Overall mean proportion of malignant neoplasms is 0.01 (95% confidence interval 0.00, 0.01). Weighted mean projected cost savings were calculated in varying formats, ranging from 6891 Euros per year within one hospital, 712 748 Euros per 10 000 patients, to 875 077 Euros per year within one country.

**Conclusion:**

A selective approach is not associated with a significant proportion of missed cancer diagnoses, and provides considerable cost savings, particularly demonstrated for cholecystectomy samples. Further discussion is required regarding how surgeons will be protected medicolegally without the safety net of routine analysis.

## Graphical abstract

Systematic review to evaluate effectiveness of cancer detection and costs with a selective approach to histopathology of all surgical specimens based on macroscopic abnormalities.

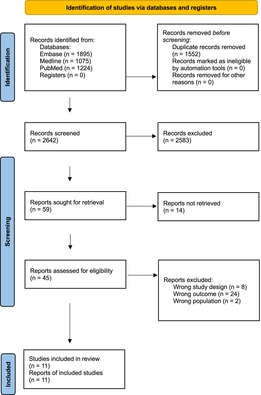

## Introduction

In most surgical services worldwide, it is accepted practice to routinely send all surgical specimens for histopathological analysis, regardless of the degree of suspicion of pathology, such as cancer, being identified. Due to increasing demands on healthcare systems and a finite supply of resources and funding, a more selective approach has been proposed, where only samples deemed macroscopically abnormal are sent for histopathological analysis. The major concern regarding a selective approach is the risk of missing the detection of malignant neoplasms, resulting in potentially detrimental patient outcomes.^1^, ^2^ However, proponents argue that the rate of detection of unexpected malignant neoplasms is extremely low and does not yield clinical significance since management would not change.^3^ Notably, the incidence of detection of gallbladder carcinoma after cholecystectomy in most countries is 0.3%–1.7%, with the vast majority (72%) of these gallbladder carcinoma cases not requiring any additional surgery or treatment.^4^ The OMEGA multicentre retrospective study analysed the surgical treatment received by 4138 patients undergoing gallbladder cancer resection across 133 centres in 41 countries.^5^ This study demonstrated that the majority of incidental gallbladder carcinomas are treated solely with cholecystectomy and that liver resection was not conclusively associated with increased survival.^5^ Furthermore, this study demonstrated that extended resections resulted in increased morbidity and mortality without clear oncological benefit.^5^ Prospective and retrospective studies have been undertaken to assess the viability of a selective approach to histopathological examination across different countries and hospital centres within those regions. This systematic review aimed to evaluate and analyse these studies outlining a selective approach, particularly for safety and efficacy in detecting neoplasms, and cost.

## Methods

A systematic review was conducted according to a protocol that was registered before commencement with the International Prospective Register of Systematic Reviews (PROSPERO, CRD42022346535). The review was reported as per the Preferred Reporting Items for Systematic Reviews and Meta‐analyses (PRISMA) 2020 and Meta‐analysis Of Observational Studies in Epidemiology (MOOSE) reporting guidelines.^6^, ^7^

### Search strategy and inclusion criteria

The population for this review was adults who had undergone any type of surgery in which a surgical specimen was sent for histopathology, in any country. The intervention was a selective histopathology approach in which surgical samples were sent for histopathological analysis based on surgeon discretion of whether cancer is suspected macroscopically. The comparator, where present (not required for inclusion), was a routine histopathology approach, where samples were sent for histopathological analysis after every surgical tissue excision. Paediatric surgical cases were excluded. The primary outcome was rate of detection of cancer while secondary outcomes were cost savings, post‐operative complications and mortality. Ovid Embase, Ovid MEDLINE and PubMed were searched between 1973 to 30 July 2022. Search terms included Histopatholog* OR “Surgical patholog* OR Histolog* OR Pathology) AND (Efficacy OR Safety) AND (Selective OR Routine) AND (Cancer* OR Oncolog* OR Malignanc* OR Neoplasm) AND (Surger* OR “Surgical procedur*” OR “Operative procedur*”). No filters or restrictions were applied.

### Data extraction

Following removal of duplicates, title and abstract screening was undertaken by two independent reviewers (ANC and LCBS). This was conducted using the web application Rayyan, Qatar Computing Research Institute, Ar‐Rayyan, Qatar.^8^ Full text screening was then undertaken by two independent reviewers (ANC and LCBS). Studies were excluded based on four categories: full text not available, wrong outcome, wrong study design and wrong population. Discrepancies were mediated by a third independent reviewer (SN). Data extraction was undertaken by two independent reviewers (ANC and MPC) using a pre‐designed template with discrepancies resolved by consensus. The extracted data consisted of study design, setting, participant characteristics, surgical intervention, number of neoplasms detected, mortality, complications and projected cost savings. This data was then synthesized via narrative and tabular methods.

### Data analysis

Data analyses were performed using Stata Statistical Software: Release 15.1 College Station, TX: StataCorp LP. The *I* ^2^ statistic was used to evaluate heterogeneity (with *I* ^2^ > 50% indicating significant heterogeneity) as was Cochran's Q P value (with *P*‐value<0.05 indicating significant heterogeneity). A random‐effects model was used throughout. A p value of <0.05 denoted statistical significance. A Funnel plot was presented for Forest plots with 10 or more studies to test for publication bias, and an Egger's Test was performed to test for small study effects. A variable was included in the analysis if at least two studies involved had sufficient values for that variable. Analyses evaluated a selective histopathology approach for mortality rate, proportion of females, proportion of males, proportion of malignant neoplasms (overall and by operation type (Cholecystectomy versus Non‐cholecystectomy) and then by region (Europe versus Non‐Europe)). Weighted means (in Euros) were calculated for projected cost saving per 10 000 patients, per year for hospital and per year within one country, since these were the different formats presented in the studies.

## Results

**Fig. 1:**
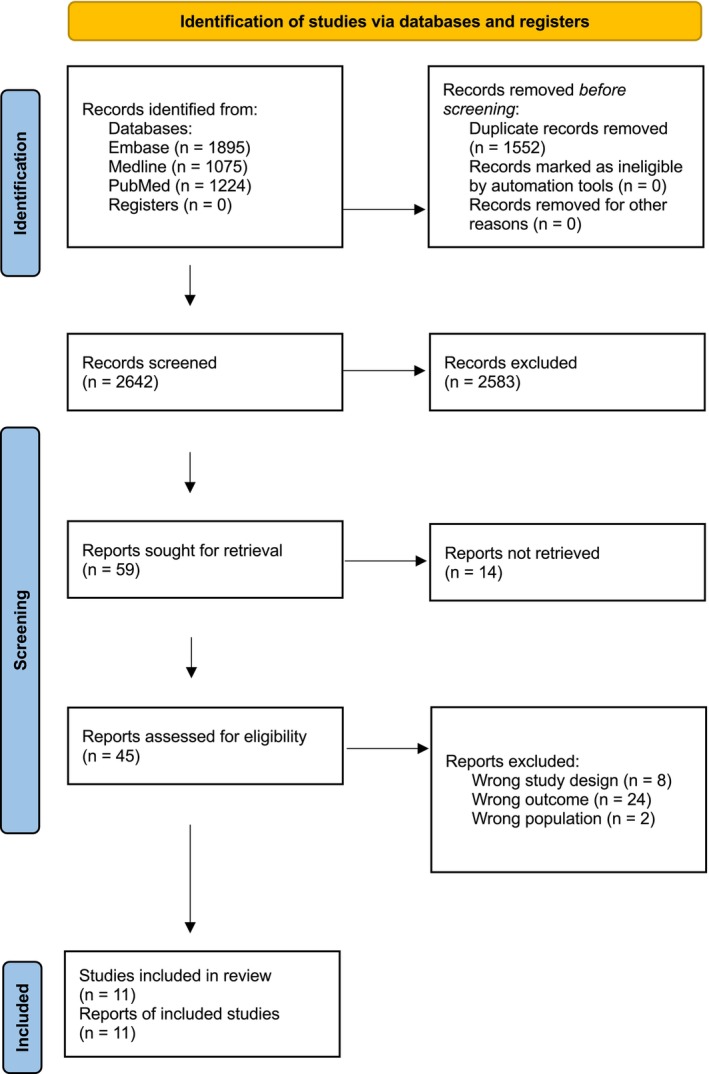
PRISMA diagram detailing the inclusion and exclusion of articles. Flowchart diagram illustrating the categorisation of records sought from databases and registers. ‘*N*=’ refers to number. This diagram depicts the total number of records identified, the included and excluded records, as well as the reason for exclusion.

The search strategy returned a total of 4194 results. Duplicates were removed, leaving 2642 studies for title and abstract screening. Of these, 59 studies were selected for full text review. There were 14 studies excluded since full text was not available. These were unable to be retrieved due to not being available through the institutional log ins of the authors. There were 11 studies which met the inclusion criteria and progressed to risk of bias critical appraisal and data extraction, and were included in the systematic review. See Figure 1.

### Study characteristics

The characteristics of the 11 included studies are displayed in Table 1.^9^, ^10^, ^11^, ^12^, ^13^, ^14^, ^15^, ^16^, ^17^, ^18^, ^19^ Six of the included studies were retrospective in design, while five were prospective. The countries of origin for the studies widely varied and were classified as Europe or non‐Europe with eight being Europe and three non‐Europe. Each study reviewed a single type of operation, with cholecystectomy being the most common with eight out of 11. The entire sample size across all 11 included studies was 26 126 patients, with the largest sample size in one study being 10 041 patients. Weighted mean projected cost savings were calculated in Euros, albeit with different parameters, such as per 10 000 patients, per year within one hospital and per year within one country.

**Table 1:** Characteristics of included studies

Study	Country	Operation	Sample size	Number of malignant neoplasms	Mortality	Projected cost savings in Euros	Sources of funding	Risk of bias (percentage)
AbdullGaffar (2016)	United Arab Emirates	Vertical laparoscopic sleeve gastrectomy	546	0	N/A	16 891.42/year in this hospital	None	78.13
Bastiaenen (2022a)	Netherlands	Appendectomy	7339	130	1	725 400 for every 10 000 patients	Government	78.13
Bastiaenen (2022b)	Netherlands	Cholecystectomy	10 041	28	N/A	703 500 for every 10 000 patients	Government	78.13
Corten (2019)	Netherlands	Cholecystectomy	319	0	0	1 245 000/year in Netherlands	N/A	73.44
Darmas (2007)	Wales	Cholecystectomy	1452	6	3	2527.57/year in this hospital	N/A	81.25
De Zoysa (2010)	Sri Lanka	Cholecystectomy	477	4	N/A	1034.36/year in this hospital	N/A	79.69
Dix (2003)	England	Cholecystectomy	1308	5	5	3951.15/year in this hospital	N/A	75
Firat (2019)	Turkey	Cholecystectomy	1112	7	1	5000 over 1 year, 3 months in this hospital	N/A	81.25
Koppatz (2018)	Finland	Cholecystectomy	2034	10	7	500 000/year in Finland	Government and private	79.69
O'Connor (2016)	USA	Neurectomy	123	0	0	58 289.11 over 17 years, 3 months in this hospital	Private	73.44
Van Vliet (2013)	Netherlands	Cholecystectomy	1375	6	4	75 839 in this hospital over 4 years, 11 months	N/A	84.38

### Patient characteristics

The majority of patients in the total population were female, largely due to the fact that the majority of studies analysed cholecystectomy undertaken to manage cholecystitis, which is more prevalent in females than males.^20^ The majority of the total sample size across all 11 included studies accounted for patients who underwent cholecystectomy, with 18 118 of the 26 126 patients having undergone cholecystectomy. There were 7339 patients who underwent appendectomy and 546 and 123 patients who underwent vertical sleeve gastrectomy and neurectomy respectively. Age was not specified for the entire sample in most of the included studies, however the reported ages ranged from 16 to 86 years across all studies.

### Patient outcomes

**Fig. 2:**
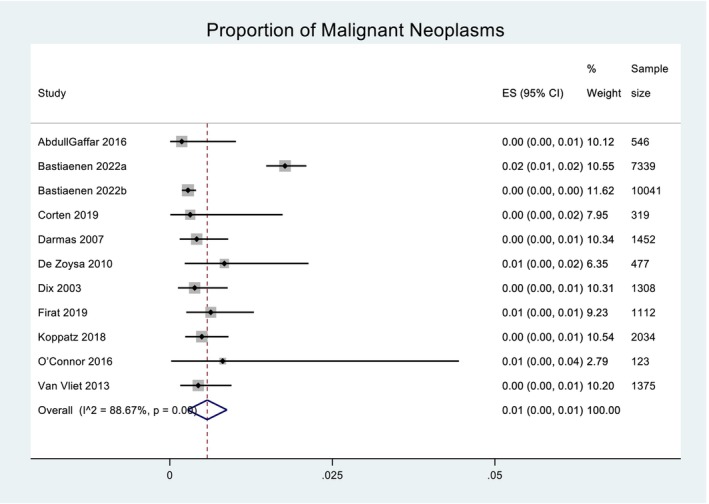
Overall mean proportion of malignant neoplasms. Forest plot demonstrating the effect size (ES) within the 95% confidence interval (CI) for overall mean proportion of malignant neoplasms with a routine versus selective policy for each of the 11 included studies. Weight of each study is listed and displayed graphically by the proportional size of each box. Total sample size for each study is listed. The red dashed line denotes the line of no effect. The diamond denotes the overall pooled effect for the studies.

**Fig. 3:**
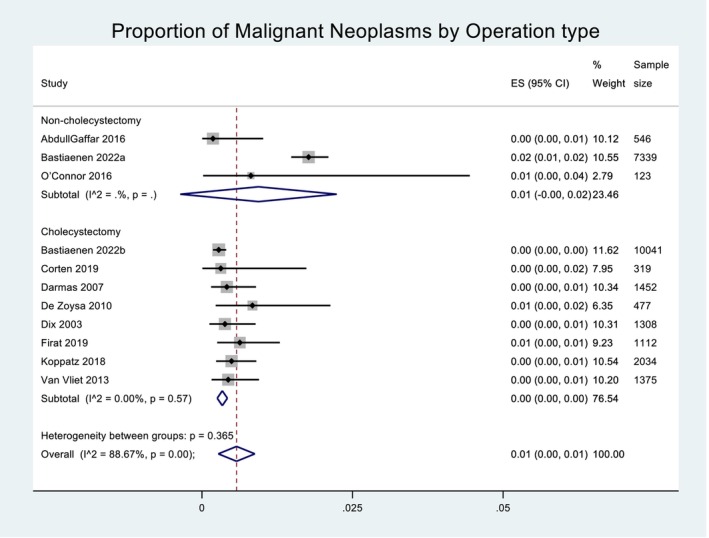
Proportion of malignant neoplasms in cholecystectomy versus non‐cholecystectomy. Forest plot demonstrating the effect size (ES) within the 95% confidence interval (CI) for proportion of malignant neoplasms by operation type. Operation type is categorized as non‐cholecystectomy and cholecystectomy. Weight of each study is listed and displayed graphically by the proportional size of each box. Total sample size for each study is listed. The red dashed line denotes the line of no effect. The diamond denotes the overall pooled effect for the studies. Heterogeneity is denoted as 0.365 which is moderate.

**Fig. 4:**
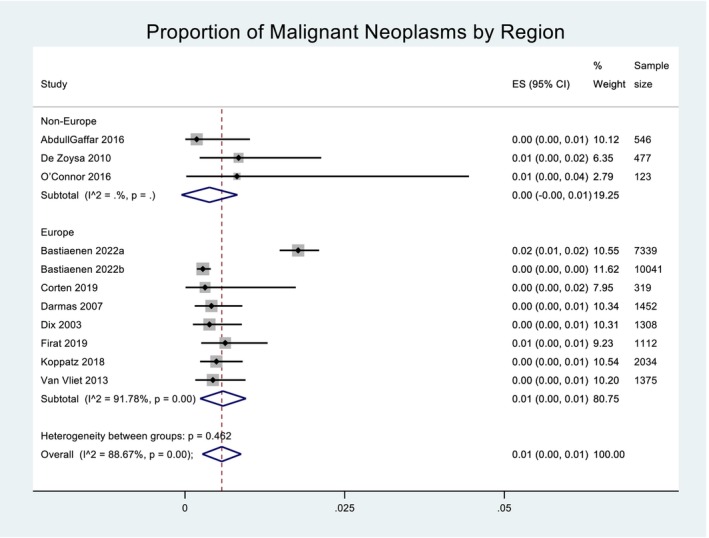
Proportion of malignant neoplasms by region. Forest plot demonstrating the effect size (ES) within the 95% confidence interval (CI) for proportion of malignant neoplasms by region. Region is categorized as non‐Europe and Europe. Weight of each study is listed and displayed graphically by the proportional size of each box. Total sample size for each study is listed. The red dashed line denotes the line of no effect. The diamond denotes the overall pooled effect for the studies. Heterogeneity is denoted as 0.462 which is moderate.

Across the 11 included studies, a total of 295 neoplasms were detected, 196 of those being malignant and 99 benign. The prevalence of malignant neoplasms was pooled across the 11 studies using a random effects model. Heterogeneity in the study estimates was assessed using the I‐squared statistic (88.67%) and Cochran's Q *P*‐value (<0.001), which showed much heterogeneity, but this was considered less relevant as a random effects model was used. The overall mean proportion of malignant neoplasms was 0.01 (95% confidence interval (CI): −0.00, 0.01. This is presented in Figure 2. A Funnel plot for proportion of malignant neoplasms suggested some degree of publication bias (Supplementary Appendix). An Egger's test did not suggest small‐study bias (*P*‐value = 0.297). When Forest plots for proportion of malignant neoplasms were presented by type of surgery, the overall proportion of malignant neoplasms for non‐cholecystectomies was 0.01 (95% CI: −0.00, 0.02) and for cholecystectomies was near 0.00 (95% CI: 0.00, 0.00), as demonstrated in Figure 3. The raw median for cholecystectomy was six, for non‐cholecystectomy was zero. The inter‐quartile range for cholecystectomy was three and for non‐cholecystectomy was 53.5. When Forest plots for proportion of malignant neoplasms were presented by region, the overall proportion of malignant neoplasms for non‐Europe was 0.00 (95% CI: −0.00, 0.01) and for Europe was 0.01 (95% CI: 0.00, 0.01), as demonstrated in Figure 4.

**Fig. 5:**
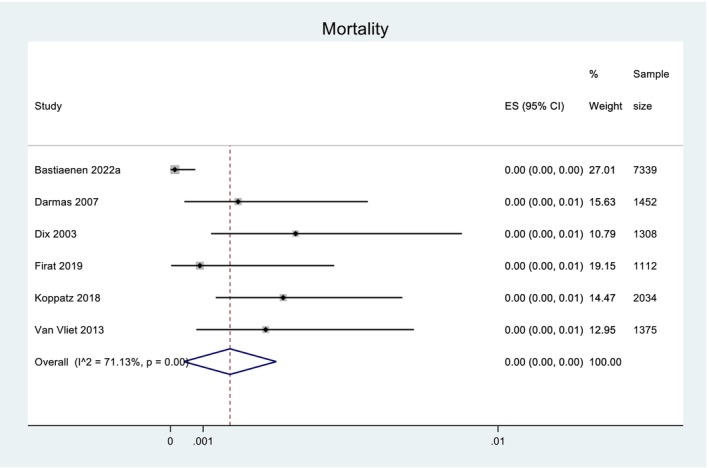
Total mortality in the studies that reported this outcome. Forest plot demonstrating the effect size (ES) within the 95% confidence interval (CI) for mortality. The six studies listed are the ones that reported mortality as an outcome, whereas the other five included studies did not. Weight of each study is listed and displayed graphically by the proportional size of each box. Total sample size for each study is listed. The red dashed line denotes the line of no effect. The diamond denotes the overall pooled effect for the studies.

Total mortality recorded across all 11 studies was 21 patients, however it is important to note that seven studies did not report mortality for the entire data set, or at all (Fig. 4). There were nine instances of post‐operative complications from the initial surgery, and 16 instances following re‐resection. Weighted mean projected cost savings ranged from 6891 Euros per year within one hospital, 712 748 Euros per 10 000 patients, to 875 077 Euros per year within one country. These figures were determined by converting the value for cost savings stated in each study into Euros from the original currency reported, and determining the weighted means in Euros using the three different methods (as listed) (Figure 5).

### Risk of bias

The Down's and Black checklist was used by two independent reviewers (ANC and MPC) to critically appraise risk of bias for the 11 included studies. Overall percentages for risk of bias for the these studies are demonstrated in Table 1. The calculated total mean was 25.09 out of 32, representing low risk of bias.

## Discussion

This systematic review demonstrated that with a selective approach to histopathological analysis of surgical specimens, the overall rate of detection of malignant neoplasms is low, which is disproportionate to the high cost associated with routine analysis. Samples that were deemed as macroscopically suspicious for malignancy were included in the ‘selective’ groups in the included studies in our analysis, that is, the samples that would be chosen to proceed to histopathological analysis with a selective approach. Notably, in the included studies, all surgical samples were sent for histopathological analysis, which enables comparison between those selected to be macroscopically suspicious and those samples that are not. With a selective policy, the weighted mean projected cost savings per 10 000 patients was 712 748 Euros,^11^, ^21^ and weighted mean projected cost saving per year per country was 875 077 Euros.^12^, ^19^ Despite a low risk of bias, significant heterogeneity was found within the included studies, particularly by region.

In most countries worldwide, all surgical specimens are routinely excised, labelled, stored appropriately and sent off to the pathology department for histopathological analysis by pathologists. Histopathological processing of surgical specimens starts with chemically “fixing” specimens usually with formalin, a substance which stabilizes cells for further processing.^22^ Fixation preserves morphology, prevents decomposition and minimizes microbial growth.^22^ Following this, tissue is embedded into a permanent paraffin wax block which is subsequently sliced into thin sections for microscopic analysis with slides which may be stained with dyes to enhance visualization of certain structures.^22^ Fixed samples allow for detailed visualization of structures, and take numerous days to process; generally up to 10 days.^22^ With the observed rates of detection of unexpected neoplasms being low across a range of different surgical specimen types, the question has been raised as to the necessity of routinely analysing all surgical samples.

Histopathological analysis not only detects the presence of malignant cells, but identifies all cells types, including benign diagnoses such as polyps and inflammation. This analysis focuses on the detection of malignancy, however it is important to note whether clinically relevant benign diagnoses would be missed with the implementation of a selective analysis policy; further analysis into this subject would be beneficial.

This systematic review categorized the surgical specimens into cholecystectomy and non‐cholecystectomy samples, since most of the included studies analysed cholecystectomies and 18 118 patients out of the total sample size of 26 126 patients (approximately 69%) underwent cholecystectomy. This operation is performed for a variety of indications, most commonly to treat acute cholecystitis. In 2016, the Dutch national guidelines were updated to recommend a selective histopathologic policy rather than routine policy.^23^ Subsequently, an analysis was conducted in a single Dutch teaching hospital into the implementation of the selective approach for cholecystectomy specimens. The study included 2271 patients who underwent cholecystectomy in a 6 year period, in which the percentage of gallbladders analysed decreased over this period from 83% to 38%, with no cases of missed gallbladder cancer in samples not sent for histopathology, and a cost saving of 65 000 Euros identified.^24^

There are several drawbacks to the implementation of a selective approach. Notably, the potential to miss histopathological diagnoses of neoplasms could have serious clinical implications. The largest study in our analysis was the FANCY study which consisted of a sample size of 10 041 cholecystectomy specimens. In this study, analysis was undertaken into whether the implementation of a selective policy resulted in missing tissue diagnoses that had clinical significance.^11^ Out of the total sample size, in the case of a selective policy, 7846 samples would not have been sent for histopathological analysis (approximately 78%).^11^ Of these, there were seven cases in which malignancy with clinical consequences would have been missed. Out of the seven patients, only two patients underwent additional surgery, the other five patients did not undergo any further treatment.^11^ The upper limit of the 95% confidence interval was 1.40 per 1000 specimens, demonstrating oncological safety with selective analysis.^11^

Significant concern is also raised regarding the medicolegal implications of adopting a selective approach due to the risk of missed cancer diagnosis. In order to mitigate this risk, we recommend guidelines are developed to streamline the process of macroscopic assessment. A prospective analysis at the large teaching hospital Máxima Medical Centre Veldhoven/Eindhoven the Netherlands demonstrated that a standardized approach to macroscopic analysis, which was established with input from surgeons and pathologists, proved easy, valid and did not result in missed gallbladder cancer diagnoses.^25^ It is imperative to include both surgeons and pathologists in the development of such an algorithm to combine the expertise of both, in order to maximize the accuracy of macroscopic analysis. The method undertaken at Máxima Medical Centre involved first irrigating the excised gallbladder with saline to remove any debris, then proceeding with thorough inspection of the surface for any ulcers, polyps, perforations, masses, indurations, calcification or focal wall thickening.^25^ From there, the surgeon incised the gallbladder longitudinally, preserving the cystic duct and gallbladder bed to the liver margin.^25^ Stones and debris were then removed to enable meticulous inspection and palpation of the mucosal and serosal wall for the aforementioned abnormalities.^25^ The surgeon then recorded his/her conclusions and decided whether further histopathological analysis was necessary, and if there was any doubt, histopathological analysis was advised.^25^ A similar approach could be implemented in other centres and countries worldwide.

Another aspect to consider is whether the experience of the surgeon has an impact on the accuracy of macroscopic analysis of surgical samples. In the FANCY study, the number of patients with missed malignancy with clinical consequences was not significantly influenced by assessor being surgeon, both, or resident; with a mean difference of 0.00045.^11^ Additionally, in this study there was no difference in the proportion of specimens sent for histopathological analysis between surgeons and residents.^11^ Regardless, we propose that a specific guideline is followed when performing macroscopic analysis to eliminate personal bias and create a streamlined approach. We also propose that further studies compare these factors to obtain more data regarding this and validate these findings. Another proposed safeguard that could be implemented is to store surgical specimens deemed macroscopically negative for malignancy for a set period of time post‐operatively. This would enable specimens to be processed if there was clinical concern developed for malignancy subsequent to the operation and recovery period.

It is important to note this analysis has some limitations. Although all studies combined consisted of a large sample size, there were only 11 studies found to be relevant for this review. Additionally, the studies varied in quality, with some being prospective multi‐centre studies involving thousands of patients and others being retrospective cohort studies involving less than one hundred patients. The studies mostly lacked a comparator group of routine histopathological analysis, meaning that clear conclusions regarding the outcomes associated with a selective approach, relative to a routine approach, were unable to be drawn.

Due to this lack of clear comparisons and substantial heterogeneity across the included studies, formal comparative meta‐analysis was unable to be conducted, and instead analyses summarizing effect sizes were pursued. However, these data provide the basis for a randomized study comparing routine histopathology and selective histopathology, which could be undertaken across multiple centres over several years. If this type of study were to be undertaken, it would provide substantial evidence to support the conclusion that selective histopathology is oncologically safe.

Notably, for non‐cholecystectomy operations in this study, the results were biased by heterogeneity in reporting, operations, and patient populations. It is difficult to draw definitive conclusions from heterogeneous studies of varying quality. Considering this, and that the vast majority of the sample size and total number of studies in this systematic review analysed cholecystectomy specimens, this review cannot form definitive conclusions regarding the non‐cholecystectomy operations, that is, appendectomy, vertical laparoscopic sleeve gastrectomy and interdigital neurectomy. It is possible that standardized approaches could be developed for the analysis of these types of surgical specimens as well as all other types. However, further review into non‐cholecystectomy operations with analysis of multiple valid papers with significant samples sizes and standardized reporting methods would be required to establish such conclusions. This systematic review is also limited in terms of population scope, since data from countries in the developing world is absent.

We propose an algorithmic approach for determining suspicion of the presence of cancer to protect patients and achieve the best clinical outcomes. Such an approach would consider clinical suspicion, radiological suspicion, and pathological suspicion to deem whether samples should be sent for histopathological analysis. For example, in cholecystectomy patients, clinical factors to consider would be jaundice, unexplained weight loss, or the presence of underlying conditions such as primary sclerosing cholangitis.^26^ Radiological features on Computed Tomography include a polypoid mass within the gallbladder lumen, focal wall thickening, diffuse wall thickening or a mass replacing the gallbladder and signs observed on Magnetic Resonance Imaging or Magnetic Resonance Cholangiopancreatography include early and prolonged enhancement and irregular enhancement in malignant gallbladder wall thickening.^26^ Pathological features include polyps, mucosal irregularities and porcelain gallbladder.^25^

## Conclusion

This systematic review indicates that the implementation of selective histopathological analysis, instead of routine histopathological analysis, is likely to be safe and effective in detecting the relatively low rate of cancer that is found in cholecystectomy specimens.

With the burden of the ageing population and chronic disease causing significant strain on healthcare systems worldwide, effective distribution of resources and funding is imperative. It is essential to continue to evaluate our processes and methods across all areas within the healthcare system to increase efficiency and re‐direct funding where deemed appropriate.

All included studies demonstrated no clinically significant outcome or risk discrepancy with the use of a selective policy. Additionally, significant cost and resource sparing was projected, which notably could assist in reducing the burden on healthcare systems and allow resources to be re‐distributed to other areas of need such as chronic disease management. Further analysis is required to definitively determine the viability of a selective approach for non‐cholecystectomy specimens given the low volume of non‐cholecystectomy studies which met the inclusion criteria for this review. Further issues to be explored include how implementing a selective policy could be streamlined in order to protect surgeons and hospitals medicolegally, due to the potential implications and protections associated with a missed cancer diagnosis, when it does, albeit rarely, occur.

## Author contributions

**Angelique N. Camilos:** Conceptualization; data curation; formal analysis; investigation; methodology; project administration; validation; visualization; writing – original draft; writing – review and editing. **Leo C. Bowley Schubert:** Data curation; formal analysis; investigation. **Marcela P. Castro:** Data curation; formal analysis; investigation. **Silas D. Nann:** Data curation; formal analysis; investigation. **Suzanne Edwards:** Formal analysis; investigation; resources; software; visualization; writing – original draft; writing – review and editing. **Brandon Stretton:** Conceptualization; methodology; writing – review and editing. **Aashray K. Gupta:** Conceptualization; data curation; methodology; project administration; writing – review and editing. **Joshua G. Kovoor:** Conceptualization; data curation; methodology; project administration; writing – review and editing. **Matthew Marshall‐Webb:** Validation; writing – review and editing. **Guy J. Maddern:** Supervision; validation; writing – review and editing.

## Conflict of interest

None declared.

## Supporting information


**Data S1.** **Doc S1**. Papers excluded due to full text not available.

**Figure S1.** Funnel plot for publication bias relating to outcome of proportion of malignant neoplasms.
