# Supplementary material for: Safety and cost of selective histopathological analysis for detecting cancer in surgical specimens: a systematic review
Source: ANZ J Surg. 2025 Jan 6;95(1-2):47–55. doi: 10.1111/ans.19380 (PMC11874888; doi:10.1111/ans.19380)
Supplement: Supplementary file 1 — Data S1. Doc S1. Papers excluded due to full text not available. [file ANS-95-47-s001.docx]

***Figure S1. Funnel plot for publication bias relating to outcome of proportion of malignant neoplasms***

Funnel plot demonstrating the prevalence against the standard error of prevalence. Asymmetry likely represents heterogeneity in study design.

***Doc S1. Papers excluded due to full text not available***

1. Bastiaenen V, Allema W, Klaver C, Tanis P, Bemelman W. Routine histopathological examination of appendectomy specimens: A systematic review. Colorectal Disease. 2018;20(Supplement 4):55.
2. Bastiaenen VP, De Jonge J, Corten BJGA, De Savornin Lohman EAJ, Kraima AC, Swank HA, et al. Routine histopathological examination following appendectomy is unnecessary: Results of the multicentre prospective fancy study. United European Gastroenterology Journal. 2020;8(8 SUPPL):845.
3. Bastiaenen VP, Van Vliet JLP, De Savornin Lohman EAJ, Corten BJGA, De Jonge J, Kraima AC, et al. Selective histopathological examination following cholecystectomy is oncologically safe: Results of the multicentre prospective fancy study. United European Gastroenterology Journal. 2020;8(8 SUPPL):90.
4. Corten B, Leclercq W, Roumen R, van Zwam P, Slooter G. A Single Center Evaluation on Implementation of Selective Pathologic Examination of the Gallbladder. European Journal of Surgical Oncology. 2020;46(2):e121.
5. Corten B, Leclercq WKG, Roumen RMH, Van Zwam PH, Slooter GD. A single center evaluation on implementation of selective patholo gicexamination of the gallbladder. United European Gastroenterology Journal. 2019;7(8 Supplement):301.
6. Echelard P, Collin Y, Geha S. Demographic, pre-operative, and grossing parameters can efficiently triage gallbladder resected for non-neoplastic disease for pathological analysis. Modern Pathology. 2020;33(3):1699-700.
7. Fanshawe A, Goodall R, Coughlan C, Sheth H. Is the routine histopathological analysis of all gallbladders justified? HPB. 2018;20(Supplement 2):S712-S3.
8. Gong Y, Garcia-Buitrago M, Rojas C, Milikowski C, Kashikar N. Is routine histologic examination of gallbladders necessary? Laboratory Investigation. 2017;97(Supplement 1):444A.
9. Gusho CA, Blank AT. What Is the Clinical Impact of Sending Tissue for Histopathology During Surgery for Known, Diffuse Metastatic Disease to Bone? Anticancer Res. 2021;41(5):2473-6.
10. Nair M, Gopinath KS, Amerendra S, Swamy S. Intra-operative frozen section analysis in breast conserving surgery: Cliche vs prerequisite? Indian Journal of Surgical Oncology. 2019;10(SUPPL 2):S111.
11. Savjak D, Medenica M. [Correlation between the clinical and pathohistologic diagnosis in "small biopsies" of the lung]. Med Pregl. 1998;51(9-10):431-5.
12. Warren BF. Pathology of the gall bladder. CPD Bulletin Cellular Pathology. 2001;3(2):103-5.
13. Wilczyńqski JR, Nowińska A, Szpakowski M, Nowak M, Szpakowski A, Władziński J, et al. [Laparoscopic treatment of benign ovarian tumors]. Ginekol Pol. 2006;77(1):40-7.
14. Zaw A, Khan FA, Ramach, ra P, Krecioch P, Anewenah L. Is routine histopathology necessary for all gallbladder specimens? Surgical Endoscopy and Other Interventional Techniques. 2018;32(1):S156.
