# Supplementary figures and images for: Safety and cost of selective histopathological analysis for detecting cancer in surgical specimens: a systematic review
Source: ANZ J Surg. 2025 Jan 6;95(1-2):47–55. doi: 10.1111/ans.19380 (PMC11874888; doi:10.1111/ans.19380)

Funnel plot with pseudo 95% confidence limits

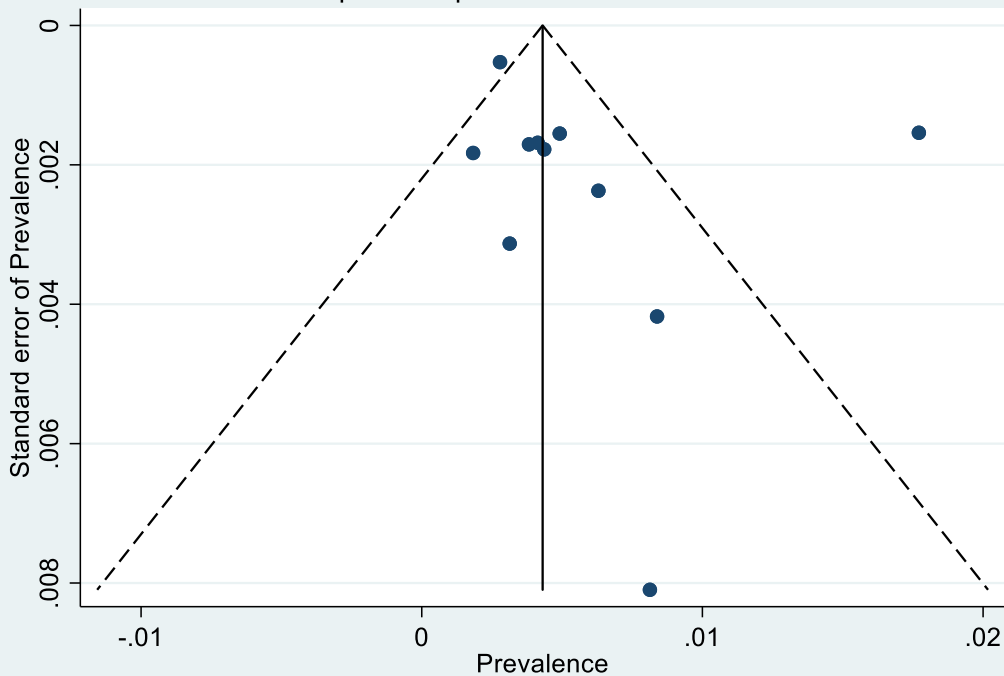

Supplement: Supplementary file 2 — Figure S1. Funnel plot for publication bias relating to outcome of proportion of malignant neoplasms. [file ANS-95-47-s002.pdf]
